# Mr. Davies's Cases of Apoplexy

**Published:** 1802-01

**Authors:** H. Davies

**Affiliations:** Piccadilly


### *To the Editors of the Medical and Phyfical Journal.*

"Humanum eft nefcire, et errare."

GENTLEMEN,

YOUR laft Journal prefented us with a ftatement of a cafe of *Apoplexy*, which, it feems, excited no fmall degree of altercation between the medical practitioners in attendance. We lament that men of fcientific minds, and liberal fentiments, fhould fo far mifunderftand or oppofe each other, as to render an appeal to the medical world neceffary; but when, for reputation's fake, fuch an appeal becomes *indispensable*, we hope, from the collifion of fentiments difcovered on the nature and treatment of the difeafe in queftion, other practitioners may be induced, with more accuracy, to inveftigate its caufes, in order more fuccefsfully to effectuate a cure.

It fcarce needs be obferved, that *apoplexy* ranks amongft the moft formidable in the dread catalogue of difeafes to which the human frame is incident; therefore, when the cafe is a *decided* one, a hefitancy of opinion refpecting the mode of treatment may be attended with the moft ferious confequences, as to the fate of the patient, and the reputation of the practitioner. It becomes then highly neceffary to have as clear a conception of the nature of the malady as poffible, although we have to lament, that, not unfrequently, the moft judicious plan, conducted by the ableft in the profeffion, proves abortive.

*Apoplexy*, with great juftnefs, I think, has been divided into two claffes, the Sanguineous, and Serous; the firft, occafioned by extravafation of blood, the other by exudation of lymph, or ferum, in the ventricles of the brain; *both* compreffing the origin of the nerves, and producing, more or lefs, a derangement of the vital functions, and lofs of voluntary motion. But as the temperature of the fubjects which give rife to thefe exciting caufes vary, it remains to be enquired, how far a fimilar treatment is likely to fucceed in both.

Not to enter into any thing difputatious on the fubject, I beg leave to recite two cafes which fell under my own obfervation within the courfe of a few months; the one I would call *sanguineous*, the other *serous*: And, when I have mentioned the plan of treatment in each, I leave the reader to form his own judgment. A lady, about fifty-three years of age, of a nervous habit, who had been accuftomed to head-achs for many years, went one day to vifit a friend at a fmall diftance from her habitation, and was feized with a flight affection of the head during her continuance there, which gave fome alarm, but but which in a little time fubfided, and fhe was enabled to walk back again to her own houfe; fhe ate her dinner as ufual, and went to repofe herfelf in bed; in a fhort time afterwards, fhe made a fignal for fome part of the family to come up ftairs; when fhe was found in a torpid ftate, hardly able to defcribe her feelings. I was fent for immediately, and on my arrival found her incapable of articulating; her pulfe was rather flow, and her breathing fomewhat laborious. I ordered her a medicine for the prefent, and unlefs any favourable alteration fhould take place in two or three hours I recommended a phyfician to be called in, which was accordingly complied with. The cafe now appeared to be a *confirmed apoplexy*. She was ordered to be cupped, and ten ounces of blood were drawn from the temples. A large blifter was applied to the fternum, acrid clyfters were adminiftered, and a volatile draught attempted to be given every three hours. But as the mufcles of deglutition were confiderably affected, and fwallowing became extremely difficult, we were under the neceffity of defifting from all attempts to adminifter any thing *internally*, and to confine ourfelves alone to outward applications. Stimuli, fuch as volatile alkali, &c. were applied to the furface of the body, and likewife finapifms to the feet. The clyfters were repeated, but without effect. She continued in this ftate of torpor from Thurfday afternoon till the following Sunday night, when death clofed the fcene. Permiffion was obtained to open the head, when about three ounces of extravafated blood were found in the *left* ventricle of the brain, and nearly an ounce of ferum in the *right*; which more than fufficiently accounted for the train of formidable fymptoms preceding her diffolution.

The other cafe I alluded to, was that of an elderly man between fifty and fixty; of a pale, phlegmatic conftitution, remarkable for obefity, which had been accumulating for a number of years, infomuch as to render it extremely burdenfome to him, and incapacitating him from bodily exercife for fome years previous to his laft illnefs. He became exceedingly debilitated at length, and was confined to his bed. In this fituation I was defired to vifit him. There was no *organic affection* manifefted when I firft attended him, but general debility feemed to prevail. An eruption of the eryfipelatous kind appeared in fome parts of the body, which put on fomewhat of a livid appearance. The indication here, I thought, was plain and obvious. To fupport the fyftem by a courfe of fuitable tonics, attending to the ftate of the bowels, and recommending a nourifhing diet, appeared to me the moft rational plan of treatment. This was perfevered in for about a fortnight, but I am forry to fay, without any manifeft advantage; the appetite became more

 and and more impaired, the patient difcovered a *comatous* difpofition, unwilling to be difturbed and fomewhat unable to fpeak. The cafe now wore an unpromifing afpect. The friends became anxious; and to afford them every poffible fatisfaction, I recommended the fame Phyfician as in the former Cafe, who was accordingly called in. From the general appearance of the fymptoms, he pronounced the difeafe *Apoplexy*. Accordingly, twelve ounces of blood were ordered to be taken from the arm, a large blifter to be applied between the fcapulæ, and a purging medicine adminiftered every two hours till it operated. In the evening of the fame day, the attendants perceived an alteration in the patient, not of the moft favourable tendency; for although he feemed in fome degree relieved for a little time, yet, afterwards, he funk gradually lower and lower; colliquative fweats came on, the extremities began to grow cold, and about eleven or twelve o'clock at night he imperceptibly fell into the arms of Death, without a convulfive ftruggle or a groan.

In neither of thefe cafes did the ftomach feem to be affected; and if it had, I fhould have confidered it not as the *primary* caufe of the difeafe, but fymptomatic, by means of nervous confent, and therefore fhould have judged the exhibition of an *Emetic* an injudicious if not a dangerous remedy. How the doctrine could be advanced by a phyfician of the late Doctor Fothergill's celebrity, I confefs I am at a lofs to account for, therefore I muft fubmit it to thofe who are far more capable of reafoning on his Theory than myfelf.

I forbear making any further Comments, contenting myfelf with relating the above Cafes juft as they occurred. If the recital fhould tend to induce other practitioners to reflect any further light on a difeafe fo tremendous in its nature, and generally fatal in its termination, I fhall feel myfelf highly gratified; although it will be readily granted, that our hopes of fuccefs are neceffarily lefs fanguine in the treatment of this difeafe, than in moft others to which the human fyftem is fubject.

I am, yours, &c.

*Piccadilly, Dec.* 10, 1801. H. DAVIES.

